# Perspectives of Dental Stakeholders on the Clinical Use of Silver Diamine Fluoride in Australia for Caries

**DOI:** 10.1177/23800844251362108

**Published:** 2025-09-04

**Authors:** S. Tang, G.H. Soares, S. Sethi, D.G. Haag, L. Jamieson, B. Poirier

**Affiliations:** 1 Adelaide Dental School, University of Adelaide, Adelaide, SA, Australia; 2 Australian Research Centre for Population Oral Health, Adelaide Dental School, University of Adelaide, Adelaide, SA, Australia

**Keywords:** community dentistry, dental public health, preventative dentistry, public health, qualitative, silver diamine fluoride

## Abstract

**Introduction:**

Silver diamine fluoride (SDF) is an effective cariostatic agent that has reemerged globally in recent years. Its resurgence is timely as dental caries remains prevalent, necessitating an affordable and simple treatment option. Its implementation in Australia has been slow, with inconsistent views on its use among practitioners, but is likely imminent given the recent global uptake. There is currently no literature examining Australian dental stakeholder views on SDF’s implementation.

**Objectives:**

This study aims to investigate Australian dental stakeholder views on the use of SDF and to identify the challenges and facilitators to its implementation for managing dental caries.

**Methods:**

Qualitative semi-structured interviews were conducted with Australian dental stakeholders. The analysis was carried out using Braun and Clarke’s reflexive thematic analysis framework and NVivo14 software.

**Results:**

Ten participants were interviewed (7 dentists, 2 hygiene therapists, and 1 hygienist) from 4 Australian states. The barriers and facilitators to Australia’s use of SDF were found within the themes of policy, research, manufacturer, teaching and exposure, and clinical setting. Major barriers to SDF adoption include insufficient training in the undergraduate setting, lack of a standardized Australian guideline detailing use, no Therapeutic Goods Administration approval for caries, and the manufacturer’s product design. Facilitators included continual professional development on SDF, manufacturer marketing of the product, and ample research supporting SDF’s effectiveness for dental caries.

**Conclusions:**

This study highlights both barriers and facilitators to SDF use in Australia, providing insight into factors that may influence its future integration into dental practice.

**Knowledge Transfer Statement:**

The results of this study show that successful implementation into Australia will require effort and changes by policymakers, universities, and manufacturers to support clinician use.

## Introduction

Dental decay remains one of the most widespread global conditions, with a 46% increase in prevalent cases since 1990 (Qin et al. 2022). Around the world, there were 3.09 billion new cases of untreated dental caries in permanent teeth in 2019 (Qin et al. 2022). The experience of dental caries leads to a poorer quality of life, due to pain and reduced esthetics that affect social interactions (James et al. 2023). It also leads to increased financial costs, missed school and workdays, inadequate nutrition, and hospitalization (Jamieson et al. 2023). Within Australia, a third of Australian adults aged 15 y and older were found to have untreated decay (Brennan et al. 2019). Many factors are associated with higher levels of untreated decay, including fewer years of schooling, uninsured groups, Indigenous identity, non–capital city locations, eligibility for public dental care, and problem-based dental visit patterns (Brennan et al. 2019). One in 4 Australians reported avoiding or delaying a visit to the dentist due to costs (Brennan et al. 2019). The provision of care in Australia is largely within the private sector, with eligibility for public dental services based on socioeconomic status and often subject to long wait times (Schwarz 2006). With the disparities in access to basic dental care (Ju et al. 2022), it is pertinent to investigate other deliverable caries management options that could be incorporated into interventions.

Silver diamine fluoride (SDF) is a topical agent with antibacterial and remineralization effect, shown to be effective in managing caries (Chu et al. 2002; Hafiz et al. 2022) and hypersensitivity (Chan et al. 2024; Zheng et al. 2022) in the primary and permanent dentition. A 38% liquid solution contains 255,000 ppm silver and 44,800 ppm fluoride ions and ammonia (Zheng et al. 2022). It is regarded as easy to use, cheap, and minimally invasive (Hafiz et al. 2022). SDF can be used to arrest dental decay by applying it to the lesion without removal of caries. This is relatively painless and fast, requires minimal resources and operator skills (Hafiz et al. 2022), and is particularly suitable in circumstances in which traditional restorative efforts are difficult.

In 2021, the World Health Organization (WHO) included 38% SDF in its essential medicines list for its use for caries management, therefore recognizing SDF to be able to meet basic health care needs (WHO 2023). However, globally, there are inconsistent policies and guidelines regarding the clinical use of SDF (Gao et al. 2021). In the United States, the Food and Drug Administration (FDA) approved SDF in 2014 for use to relieve dentinal hypersensitivity (Gao et al. 2021). Two years later, SDF was granted breakthrough status by the FDA for dental caries, as a sign of its commitment to assess SDF for caries use (Gao et al. 2021). More recently in 2022, non–dental health professionals were granted approval by the American Medical Association to use SDF to manage dental caries (Botko 2022). During the COVID-19 pandemic, SDF was highlighted as a useful dental treatment for dental caries, when aerosols generated during traditional treatment methods posed a danger for viral transmission (Eden et al. 2020). There has recently been a renewed interest in SDF globally (Gao et al. 2021). However, adoption has been limited, possibly due to the absence of universal guidelines and inconsistencies in clinical and didactic teaching programs across universities (Gao et al. 2021).

There has been varied use of SDF in Australia across time. While the use of SDF has been considered limited by dark discoloration occurring upon application on carious areas, subsequent potassium iodide (KI) application was demonstrated to reduce staining as it precipitates the free silver ions (Patel et al. 2024). Staining on soft tissue is temporary, as the silver does not penetrate the dermis and will be shed with the keratinocytes (Crystal et al. 2017). Another consideration is the inclusion of ammonia in SDF, which results in a high pH that may cause soft-tissue burns on contact and produces an unpleasant smell and taste (Zheng et al. 2022). Hence, a 40% silver fluoride (AgF) formulation, in which ammonia-associated disadvantages (Zheng et al. 2022) are eliminated, was effectively used for caries management by Dr. Graham Craig at the University of Sydney in the 1980s (Craig et al. 1981). However, there is limited global use of silver fluoride formulation and high-quality research on its effectiveness. On the other hand, a systematic review found SDF achieved an 81% caries arrest rate in children after 12 mo (Gao et al. 2016).

The Australian Therapeutic Goods Administration (TGA) has approved SDF and AgF to treat dentinal hypersensitivity, while the Australian fluoride guidelines recommend the use of SDF or AgF to manage caries when traditional approaches are not possible (Do and Australian Research Centre for Population Oral Health 2020). SDF is also included in the Australian Schedule of Dental Services and Glossary 13th edition under item code 123 to be used as a cariostatic agent (Australian Dental Association 2022). The high prevalence of untreated dental decay calls for a consideration to all caries management options available to improve access to care (Qin et al. 2022). SDF is effective in arresting dental decay (Chu et al. 2002; Hafiz et al. 2022) and is a viable option as it is inexpensive and easy to apply, making it suitable in populations with reduced access to dental care, whether due to financial, physical, behavioral, or other reasons. The agent’s implementation into Australia is imminent, with the growth in global activity surrounding SDF and Australia’s need for an effective and affordable options for managing dental decay. Despite its long history in Australia and effectiveness demonstrated by several experimental studies, broader implementation has been slow with inconsistent use. Currently, there are no studies examining Australian dental stakeholder views on SDF implementation, which would be highly useful in guiding successful inclusion of SDF into Australia’s health care system. The aim of this qualitative study is to understand Australian dental stakeholder perceptions on the use of SDF and the challenges and facilitators for wider implementation in Australia.

## Methods

### Positionality Statement

The primary author is a final-year dental student from Australia who has previously worked with silver fluoride in pediatric clinics and attended university classes on SDF use. As a student interviewer, limited experience in clinical practice and caries management may present a challenge in fully grasping the depth of the interview responses. Completing student placements within the public system may lead to a preconceived perspective on the role of SDF in oral health, as it is primarily viewed through the lens of public health care. The supporting team consists of clinicians and researchers from Brazil, Australia, New Zealand, and Canada. We come to this work with shared values of health equity and a desire to progress public health dentistry.

### Study Design

Qualitative semi-structured interviews were used to investigate dental stakeholder opinions on the use of SDF. This included a flexible question guide with open-ended questions to gather deep, insightful perspectives on SDF (Jamshed 2014).

Questions asked of stakeholders related to views of guidelines, regulations, and teaching of SDF and the challenges and facilitators for its implementation in Australia (for the full interview guide, see Appendix 1). Perspectives on AgF were also accepted if offered by the interviewee due to the similarity in its implementation and use.

The semi-structured interviews were carried out between August 2023 and March 2024 concurrently with the ongoing recruitment process. The interviews were conducted at a time and place best suited for interviewees, with each session running between 30 min to 1 h. This included face to face at the University of Adelaide, a café, or dental clinic or online conferencing via Microsoft Teams. Audio recordings of both in-person and online interviews were obtained for transcription and analysis. There were no video recordings. All interviews conducted online were automatically transcribed with Microsoft Teams, and audio recordings of in-person interviews were transcribed by the interviewer. All transcriptions were reviewed in depth by the interviewer (S.T.) for any errors in transcription and as part of the data familiarization process. Participant details and any identifying information were removed from the transcripts prior to analysis.

### Interview Guide and Development

The interview guide was created by S.T. to facilitate discussion of the participants’ demographics and their experience with SDF, including education received and perceived barriers and facilitators to its use in Australia. All researchers then played a role in reviewing the content and refining the clarity of the questions. The guide served as the foundation, enabling follow-up questions to further explore responses and uncover emerging ideas during the interview.

### Ethics

The project received ethics approval from the University of Adelaide Human Research Ethics Committee (H-2023-1692). There was confidentiality and informed written consent for all participants.

### Participants and Recruitment

The inclusion criterion for participation in this study was being a dental or oral health professional focused on clinical, policy, or research fields. There was a primary focus on the public clinical setting, but we also included private, urban, and rural contexts. Participants had to be older than 18 years and fluent in English (Table 1).

**Table 1.** Participant Inclusion and Exclusion Criteria.

Inclusion criteria: • Clinicians in dentistry between the ages of 18 and 70 y, residing in Australia • Participants with prior experience of at least 2 y working in oral health as dental professionals, policymakers, or researchers Exclusion criteria: • Participants with vested interest or support from silver diamine fluoride manufacturing companies in the previous 5 y were not eligible or invited to participate • Non–English-speaking participants

Key dental stakeholders were identified using purposive sampling with representation across Australian’s states and territories. Stakeholders were identified through dental networks including the Australian Dental Association (ADA) and Australia’s 9 dental schools. Their contact information was located from their official work or organization websites. Stakeholders were then contacted via email with the participant information sheet containing information on consent and confidentiality (Appendix 2). A draft of the email sent to participants is included in Appendix 3. Consent forms were signed and returned before the scheduled interview. Stakeholders were contacted mainly through snowballing techniques through dental networks and organizations. We sought to obtain representation from different geographic locations and professional perspectives. After recruiting stakeholders from more than 50% of Australian states and from various clinical and professional backgrounds, limited new knowledge was being shared during interviews. Parallel to this, engagement through recruitment channels slowed significantly; therefore, following at least 3 attempts to make contact, a decision to end recruitment was made.

### Data Analysis

The analytic process used Braun and Clark’s reflexive thematic analysis framework, in which the researcher’s unique experience was embraced and part of the process. As their contribution is unavoidable, the researchers’ assumptions and perspectives are acknowledged (Braun and Clarke 2006, 2019, 2021). Interview transcripts were refamiliarized through time spent reading and immersing. Coding of the data was conducted using NVivo 14 software (QSR International Pty Ltd. version 14.23.3) and considered the entire dataset. The codes aimed to capture the segment’s core idea and importance. After coding of the transcripts, similar codes were meaningfully grouped on NVivo and interpreted to generate themes related to the research question. Coding and theme generation were performed primarily by S.T. and reviewed and refined by all members of the research team. The themes were reanalyzed, accounting for the entire data set, and labeled. The themes were then represented on a conceptual model to understand the pattern and relationships among them. The analysis was written up to tell the story of the data, with examples chosen that best illustrate the themes. This study is reported in alignment with the Standards for Reporting Qualitative Research guidelines (O’Brien et al. 2014).

## Results

Ten interviews across 4 states in Australia were conducted with 7 dentists, 2 oral hygiene therapists, and 1 hygienist. There were 6 women and 4 men. Years of participant clinical experience ranged from 11 to 49 y (Table 2). All participants had worked in both the private and public sector at some point during their career. Nine participants had held a teaching role at a university, either in clinical training or lecturing. Six of the participants also held nonclinical roles in research or policy making. Three had a postgraduate specialist degree in pediatrics or special needs dentistry. Seven participants had used both SDF and AgF in their clinical practice, while 2 had experience exclusively with SDF and 1 solely with AgF. None received formal training on either during their undergraduate studies; however, 3 received training during their postgraduate education, while 7 pursued additional training through continuing professional development.

**Table 2.** Participant Characteristics.

	Participants (*N* = 10)
Gender	
Female	6
Male	4
Role	
Clinical	10
Nonclinical	6
Years of experience	
10–30	5
31+	5

Participants acknowledged the changing use, popularity, and availability of SDF in Australia across their careers. Barriers and facilitators were in the main 4 themes, which were policy, research, manufacturer, teaching and exposure, and clinical setting (Figure).

**Figure.**
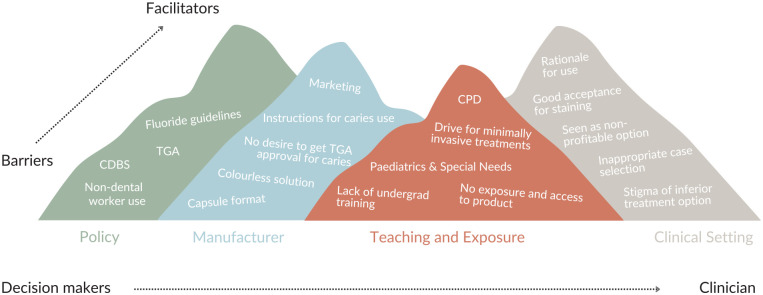
Conceptual model of facilitators and barriers to use of silver diamine fluoride in Australia.

Since many interviewees shared their views on silver fluoride, their perspectives were included in considering its broader use as a cariostatic agent in Australia.

### Evolving Perceptions and Momentum of SDF Adoption in Australia

Participants described the fluctuation in popularity of SDF and AgF since its first introduction in Australia. At times of its dwindling popularity, the product was difficult to obtain in Australia. It was rumored among dental practitioners that there was manufacturer disinterest to sell a supposedly nonprofitable product: “They weren’t interested in doing anything else with this, you know, because it wasn’t a huge marketable sort of thing” (participant 1). However, this commercial disinterest had ramifications for practitioners, and there was a yearning for the product due to a perceived great need for its use: “I missed it terribly because there was such a place for it in dental care” (participant 1). During this time, clinicians found themselves independently seeking out the product through their networks and from key Australian individuals involved in SDF production: “So we just do sort of ring [them] up and say, have you made up a batch? Yeah, they did send it over” (participant 8). Participants acknowledged the recent revival of SDF in Australia as a treatment option: “It’s had a bit of a resurgence and that [has allowed us to] kind of incorporate it back into the toolkit and use it” (participant 7).

### Policy and Guideline Tensions Hinder SDF’s Path to Wider Use

Participants named several Australian policies and guidelines that needed amendment to support wider uptake and use of SDF. One recommendation was the lack of inclusion of the item code 123 under the Child Dental Benefits Schedule, for the application of a cariostatic agent. This is important as “things that are not included or excluded which does influence the treatments that are proposed to patients or that are taken up from patients as well” (participant 7). Another suggestion was clarifying the wording of SDF use within the fluoride guidelines: “I think the fluoride guidelines itself, when it comes around to revising it, I think the wording around the use of silver fluoride needs to be strengthened” (participant 10). There was concern over the lack of an Australian-wide guideline with details of clinical use for SDF: “We have guidelines at the school level but there’s no Australian standard” (participant 4).

The TGA has approved SDF for dentinal hypersensitivity use in Australia only and not for dental caries. Participants commented on the TGA’s decision to limit SDF approval to dentinal hypersensitivity, noting that in practice, this guidance was often overlooked: “There is a certain ‘rolling of the eyes,’ I guess when you know it is only approved for dental sensitivity like. Oh, come on. Get real. . . . And everyone sort of goes wink wink” (participant 8). The TGA’s approval for its use for dentinal hypersensitivity is unpopular, as clinicians believe there are better options for managing sensitivity that do not involve staining: “Probably the staining would be a reason why I would not [use it for hypersensitivity] . . . there’s other ways that we could manage sensitivity that didn't have that that issue” (participant 9). The TGA’s approval does not appear to align with how clinicians use and perceive the SDF product.

There was also acknowledgement of the potential legal issues over use of SDF against the TGA approvals, which may stop widespread program-based use of SDF as a cariostatic product: “Very reluctant to use [SDF] for public service wide sort of delivery . . . that is not, you know, officially sanctioned as it were” (participant 6). The legal matter may also hinder educational courses on publicly speaking on SDF for caries, if the “lecturer has been saying . . . you can use it for this and that . . . she’s going to put herself open to possibly some legal exposure of going beyond the guidelines” (participant 8). This could further hinder the integration of structured education of SDF in university curricula and continued professional development courses. Clinicians felt the onus fell on them to educate themselves and the patient on the product, if using against TGA approval: “And it really comes down to the clinician to understand that it’s not approved and using an off label and following the right consent processes” (participant 4).

Participants expressed a desire for the TGA to approve SDF to be expanded and include caries arrest, as it may increase clinician confidence and use, if they feel the product has been scrutinized: “Once [TGA approval is gained] . . . it gives [clinicians] some confidence and comfort that they’re using something that presumably . . . tested really well . . . gone through with a fine tooth comb as to the potential issues” (participant 6). With TGA approval, there may be direction on creating a standardized guideline for its use.

Differing perspectives were offered on whether further research is necessary to amend TGA approval, with the argument that sufficient evidence exists but is being overlooked: “If there’s already 50/60 years of evidence out there for the caries arresting, why aren’t we looking at all of that?” (participant 3). Uncertainty remained on whether research was the main barrier to its use: “Whilst that’s important that we keep collating that and getting that research done, I don’t think that’s going to be the thing that necessarily changes people’s behavior in terms of using it” (participant 7). Participants questioned Australia’s restrictive approval of SDF compared with its broader acceptance globally with frustration over the lack of alignment with international evidence: “I don’t understand why we don’t take global evidence . . . so it’s accepted in Brazil and America [for caries] but in the UK, Australia, and I think Canada, its only desensitizing” (participant 3). Despite the substantial body of global evidence, Australia’s incorporation of this research into SDF policy and regulations remains limited.

Generally, participants were opposed to policy changes for non–dental health practitioners to use SDF for caries. The main concern was inappropriate case selection due to lack of knowledge of detection of caries and suitable treatment options. Dental clinicians were also doubtful non–dental health practitioners could accurately apply SDF without harming the gingiva:

Someone that’s never held a micro brush before going into the mouth who’s never gone into the mouth before to apply it on a lesion that they’ve never seen before is a very technically challenging process . . . also being able to differentiate caries of various severities if dentists already are getting it wrong, what’s the chance that are the health practitioners are going to get it wrong? You know very high. (Participant 4)

However, there was disagreement between participants, with few supporting its potential use by non–dental practitioners in rural and remote areas, only with further training: “In really regional remote area. . . . It’s better than nothing. Maybe” (participant 9). The policy change may also be hindered by the dentist’s desire to protect their scope of work: “It’s another . . . chip at . . . what dentists can do. Interesting” (participant 8). One participant noted that if this policy change was to be successful, the dental communities support is needed: “So if we were to implement something like this in Australia, then I think it needs to have dental completely on board” (participant 5).

### Manufacturer Design and Regulatory Action Influence Clinical Usability and Acceptance of SDF

Interestingly, manufacturers have been marketing SDF for caries arrest, despite the TGA approval for dentinal hypersensitivity. Clinicians have been guided by manufacturer instructions in the absence of formal university training, but they remain confused about the regulations behind its use:

Especially if they’re buying the product from a manufacturer, right? And if the manufacturer already selling it as a caries arrest medicament which they are. That’s what the clinicians are going to go by if they don’t know, especially if they don’t know any better. (Participant 4)

With so many clinicians using it off-label for caries, participants felt manufacturers have little incentive to seek TGA approval for this use, as there was no financial benefit:

My understanding is that the companies have to seek the TGA approval themselves and the only reason that they haven’t done it is because it’s quite cost prohibitive and the companies know that people are using it quite successfully off label and I suppose they haven’t really had a need for that motivation to seek that extended TGA approval. (Participant 7)

### Limited Training and Exposure Create Confidence Gaps in SDF Use

Participants felt there was minimal to no training on SDF during undergraduate studies, with many unaware of the product upon graduation. Participants who have held teaching positions noted the gradual increase in recognition of SDF during undergraduate degrees; however, it was inconsistent, with no standardized syllabus or clinical exposure: “There’s potential for those students that are in those clinical placements, but my understanding is otherwise no, it’s very inconsistent and it wouldn’t be across the board or a particularly regulated or followed up thing” (participant 5). A suggestion included more hands-on training for SDF in the undergraduate course: “Probably better training really and introducing it actually into that undergraduate training and in a practical way more than just one or two lectures” (participant 9). Without sufficient training and clinical exposure, clinicians were less inclined to use SDF in the workplace: “So I think that becomes a bit of a barrier that you don’t see it, you don’t use it and then it’s kind of is a self-fulfilling prophecy” (participant 7).

Clinicians were introduced to SDF through postgraduate courses, overseas exposure, colleagues, or continual professional development (CPD) programs. The special needs unit and pediatric unit were the specialties that taught SDF in their postgraduate courses and introduced SDF to the undergraduate students. Without sufficient knowledge, participants independently sought further education on SDF through CPD, online material, or through manufacturer resources: “Yeah, I’d say as a general rule, the training that I’ve received has been on my volition” (participant 7). SDF was also discovered when seeking CPD for minimally invasive techniques. Two key individuals, Dr. Geoff Knight and Dr. Graham Craig, were mentioned as highly influential in Australia’s teaching of SDF, with many attending their courses or engaging with them to seek training: “Dr. Geoff Knight from Melbourne. . . . He’s very innovative in techniques and non-invasive procedures. Minimal intervention was very much a guiding principle for me . . . I was influenced by his lectures and clinical presentations in the 90s” (participant 8). Dr. Graham Craig played a pivotal role in the adoption of AgF in Australia during the 1980s, particularly its widespread use in the public sector. Participants recognized him for both teaching and advocating for its use among dentists.

Historical success and use of the material empowered clinicians to use SDF for future cases. The importance of reviewing earlier cases was emphasized, as it reinforced belief in SDF’s effectiveness: “I didn’t see her again for 6 years and then when we saw them, they were having no issues with their tooth and we’re glad for that treatment option [of SDF]” (participant 3). There is personal reflection of rediscovery and sharing of success stories between clinicians to confirm SDF’s efficacy: “I used it in the first round. I said I’m using it again now and I am getting exactly the same results as what you are” (participant 1). These positive outcomes, both among individuals and within dental communities, bolster confidence in SDF’s effectiveness and motivate continued use.

### Clinical Practicality and Case Selection Influence SDF Uptake

There were multiple groups identified to benefit from SDF including in rural and remote communities, people with special needs, medically compromised patients, children and older adults,

where your normal sort of routine restorative measures may not be as effective . . . principally because of their child behavior . . . and that can be, in my view, at any age, whether it’s children or in adults as well, because with the elderly, for instance, who might be suffering from neurocognitive deficiencies, or people within special needs.” (Participant 6)

This included anxious patients, in whom there may be greater acceptance of care if initial treatment is less invasive: “They’ve got that element of confidence . . . after that first visit where you know the people have had a positive experience” (participant 1). There was also focus on SDF’s capacity to deliver care in aged care settings due to the quick onset of decay and limited access to care:

Patients that I’d seen for several years that ended up in nursing homes, when I next saw them they pretty much went from no caries to caries in 28 teeth, uh within 6 months of being in a nursing home, which is horrible. (Participant 3)

The most identified reason for application was the stabilization of caries and interim use before definitive treatment, which allows time for thoughtful treatment planning: “So arresting the caries, creating time to evaluate, to implement all the other preventive measures, to then look at, okay, what is the appropriate restorative management that is required?” (participant 7). This was suggested to be highly useful while waiting for treatment under general anesthetic or when current finances were limited. SDF’s place in the public sector is highlighted for its ability to stabilize decay where there are long waitlists:

The public setting with lots of patients that you could at least try and stabilize . . . the appointments take longer, they take longer to get in . . . are they going to have 10 endos by the time they actually get to the end of their treatment because things weren’t arrested soon enough. (Participant 2)

However, the lack of resources to conduct follow-up appointments in the public sector was a barrier to its use: “We don’t have the capacity to review patients after placing it, so you can’t place a top up or confirm that it actually has worked” (participant 9).

A clinical handling barrier identified was the high pH of the agent causing chemical burns to the soft tissues, which is a danger to the clinician and the patient: “Actually I know a dentist who tried it and gave himself a gingivectomy” (participant 1). The packaging of the agent was found to be an issue for control of the material: “One of the things we were worried about was perforating the—we had the little capsules perforating the seal it actually splashes out” (participant 3). It was acknowledged by participants that silver fluoride might be chosen for use to avoid this adverse effect. The handling is further made difficult since Australia’s formulations of SDF and AgF are colorless, unlike America’s Advantage Arrest, which has a blue tint: “Also being colorless. It is difficult to see where you’re applying it, and so that’s another barrier” (participant 4). There was confusion on whether the material required refrigeration, and for those who believed it did, refrigeration prevented their use for trips to rural and remote communities: “The bottle suits us much better because it doesn’t have to be refrigerated” (participant 1).

The acceptance of staining by patients was found to be high by practitioners: “I can only think from memory a handful of patients, they’ve declined the treatment. Usually, the parents are pretty accepting” (participant 9). Acceptance for staining was facilitated by good, informed consent through presenting alternative options, with patients more likely to accept SDF if the alternative was tooth loss: “In that circumstance where, you know, losing a tooth is not an option, then you know silver fluoride becomes, you know, an option for them” (participant 5). There was discussion of the recent change in cosmetic standards, which meant it was more difficult to gain acceptance for the staining in some cases: “People accepted that back then because they weren’t after white teeth” (participant 1).

SDF may be viewed as a less desirable treatment option for clinicians and patients, especially when alternate options are available, which can hinder its acceptance: “Some stigmas are related to the use of it being isolated to communities and locations where patients don’t have other access and it’s seen as an inferior treatment option” (participant 7). A shift in views on the management of caries may be necessary, to move away from solely traditional restorative care to more biological driven approaches:

I think clinicians by and large think restorations is the gold standard way of caries management. And I think that is a known issue, whether it’s implicit or explicit. I guess the way that clinicians . . . really be re-educated about how to manage care in a biological approach. (Participant 10)

There was discussion on SDF’s common alternative in children, which is the use of Hall crowns. It was often seen as a similar choice to SDF, with the same issue of poor esthetics with a metal crown but potentially more painful to use than SDF.

Appropriate case selection was emphasized as the key to successful outcomes of SDF use: “Selection was not even thought about, and so the abuse of these medicaments is a significant issue because people see it as some sort of panacea type treatment . . . we could just put it on” (participant 4). The lack of careful consideration in selecting cases has led to a misuse of the treatment. Participants noted that inadequate knowledge of indications and contraindications led to poor outcomes: “Now when you are trying to do lots of stuff with that material that you’ve got, then you run the risk of it becoming painful or people not liking it” (participant 1). This perspective highlights the need for a more thoughtful, targeted approach to SDF application, instead of viewing it as a one-size-fits-all treatment.

Participants recognized there was a place for SDF in private clinics to be used for caries on partially impacted wisdom teeth, in the elderly population, and as a financially affordable option: “You don’t have to think that hard to see it as a useful tool in your toolkit in mainstream private practice” (participant 8). Use in private clinics was hindered by availability, with clinicians hesitant to ask their bosses for the material: “I am reluctant to say ‘Ohh I want this,’ ‘I want that material’ and if no one else is using it then” (participant 8). The limited financial profitability of SDF treatment presents a significant barrier to its adoption in private practice when compared with more lucrative procedures: “Depending on what the practitioner sees, if they see, you know, high end smile design and things like that, they’re not going to be using silver fluoride” (participant 2).

## Discussion

The research aimed to discover Australian dental stakeholder views on the use of SDF and perceived challenges and facilitators to its implementation into Australia. The results showed 4 main themes of policy, manufacturer, teaching and exposure, and clinical setting. These themes tended to overlap, with policies and manufacturer decisions underpinning clinical use and teaching.

### Policy and Guidelines Tensions Hinder SDF’s Path to Wider Use

Surprisingly, clinician use of SDF for caries arrest was not hindered by the TGA’s approval of SDF for hypersensitivity. Many clinicians were comfortable using the product if they had prior experience with it and were familiar with the evidence. Supporting recommendations from guidelines also empower clinicians to apply the product despite off-label use (Crystal et al. 2020). Instead, there were concerns that the off-label status would be a barrier to developing educational courses on SDF and the use of SDF in public widespread programs (Dang et al. 2020), if the legality of the use of the product was not known. Although TGA approval may not sway a clinician’s decision on SDF use, it will inhibit its use in settings that would benefit from it most. There was a general consensus among participants that the lack of TGA approval for caries arrest was not due to the absence of evidence but more to the lack of motivation for manufacturers to seek approval. Overall, TGA approval for caries arrest would be helpful for widespread implementation in Australia, but the question is who will drive the change? Further efforts into increasing high-quality research on SDF might be futile, if the driving force required for change is in the hands of the manufacturer.

Despite the inclusion of SDF into the fluoride guidelines (Do and Australian Research Centre for Population Oral Health 2020), Australia is still missing a standardized guideline on SDF detailing its use, indications, and contraindications. Instead, clinicians are turning to the manufacturer’s instructions and resources to inform their use. Manufacturers have been promoting the agent as caries arrest, unlike the TGA approval (Patel et al. 2024). This marketing is a source of confusion for many clinicians. The TGA approval for caries arrest may allow the development of detailed Australian guidelines, which may reduce confusion and fear of legal ramifications among practitioners.

We identified resistance among practitioners to allow non–dental health professionals to use SDF for caries arrest. Although there is acknowledgment of the usefulness of this arrangement (Ruff et al. 2024), there are greater concerns of the safety for patients. With the United States making changes in this arena (Botko 2022), it is a potential next step in Australia’s implementation. However, if this were to be implemented, it would be beneficial for the dental community to be on board. There would need to be elaborate training developed by dental associations, teaching case selection and good clinical handling of the material (Ong et al. 2024). In 2023, policy changes meant Aboriginal and Torres Strait Islander health workers were able to apply fluoride varnish for caries management after completion of training (Australian Dental Association 2023). They also learned to provide oral hygiene instructions, provide dietary advice, and identify basic dental problems to guide referral to dental professionals (Ramsey 2023). This practice has been recommended since 2014 in the United States, allowing pediatric primary care providers to use fluoride varnish for caries management in children (Goff et al. 2022). A qualitative explorative study found surveyed dentists in the United Kingdom perceived SDF application as simple as fluoride varnish application (Seifo et al. 2020). It may be possible to take a stepwise approach, to gradually transition to expand the scope of practice for non–dental practitioners to use SDF.

### Manufacturer Design and Regulatory Action Influence Clinical Usability and Acceptance of SDF

The manufacturer plays an important role in SDF implementation within Australia, through product provision, marketing, and the push for TGA approval. Participants discussed the difficulties of clinical handling with the product, listing issues with its colorless solution, capsule format, and refrigeration. There are concerns of accidental spillage when piercing the capsules and further difficulty detecting the colorless solution. These issues are within the control of the manufacturer, which could be resolved to improve storage and reduce accidental staining and soft-tissue irritation.

### Limited Training and Exposure Create Confidence Gaps in SDF Use

The lack of undergraduate training and exposure was an important barrier to clinician’s use of SDF. Insufficient training is a global problem with many practitioners turning to continual professional development to increase their knowledge (Schroe et al. 2022; Chai et al. 2024). Although there is improvement in teaching and attitudes toward SDF over recent years (Dang et al. 2020), participants recognize there is still inconsistent teaching across and within Australian universities. There is no study examining the teaching curriculum of SDF across Australia at the undergraduate or postgraduate level. Training in undergraduate should include didactic material but also practical handling and use of the material under supervision (Schroe et al. 2022). This might be guided by the Australian Dental Council if included in their criteria. Facilitators of SDF use have been the CPD courses available to Australians with thanks to the key individuals advocating for SDF and AgF.

### Clinical Practicality and Case Selection Influence SDF Uptake

Acceptability of staining by patients was perceived to be high among clinicians, if care was given to explain the treatment and alternatives (Cappiello et al. 2024). The importance of communicating the treatment should be highlighted within educational materials (Sabbagh et al. 2020; Crystal et al. 2023). However, it was noted that SDF was often seen as an inferior treatment option by both patients and clinicians (Chai et al. 2024). This barrier to use indicates the need for education of its indications and usefulness and emphasis on the tertiary preventive capabilities it may have in community public health interventions (Sabbagh et al. 2020; Seifo et al. 2020). Exposure and training at an undergraduate level may help develop clinician views of the product. This must be done with care to ensure that there is still good understanding of case selection to ensure the material is not inappropriately used.

There is still resistance for uptake of SDF in the private dental setting, affected by practitioner preference, attitude, and profitability, but both sectors must play a role in delivering essential care. In 2020 in Australia, 83% of employed dentists worked in private compared with the 4.9% who worked in the public sector (Australian Institute of Health and Welfare 2023). There is a disproportionate number of dentists employed in public compared with the number of patients who require care in the public system (Brennan et al. 2008). A clear division between private and public blurs the role each practitioner has in addressing the large inequity in dental care that exists. The government may wish to investigate programs or funding schemes to allow this to occur. As SDF is affordable and quick to apply (Seifo et al. 2020; Chai et al. 2024), this may be a practical option to include in dental schemes accessible by both sectors for specific cases.

### Future Directions

Management of dental caries has traditionally been restorative and remains the gold standard treatment. However, access to this clinical management option is not the reality for many. Use of SDF may not provide function to the tooth, like restorative care can, but it will arrest the decay and prevent further complications (Hafiz et al. 2022). There needs to be a shift in the current way we think about dentistry. Many participants identified SDF as an inferior treatment option, which, compared with restorative care, may be the case, but many Australians are facing difficulties accessing this option (Brennan et al. 2019; Ju et al. 2022). The goal is to improve access to dental care to meet the demands of groups who need it most. First, TGA approval is a big step in removing the regulation barrier to allow the development of programs, guidelines, and education to allow use of SDF in community settings. Second, oral health should be integrated into mainstream health systems to allow broader delivery of nonspecialized services such as application of SDF. A good first step would be the use of non–dental health professionals to apply SDF for caries management.

The study also highlighted clinicians’ experiences and knowledge of silver fluoride, likely due to Australia’s long history with the agent, particularly through Dr. Graham’s work in Sydney (Craig et al. 1981). Despite its limited popularity in the global literature, silver fluoride is available in multiple formulations in Australia and might be considered for broader implementation, alongside SDF. This may be hindered since there remains limited high-quality evidence on silver fluoride’s effectiveness across different population groups and settings. Furthermore, while interviewees recognized its clinical handling advantages, they saw no significant differences from its broader implementation into Australia when compared with SDF. This highlighted that the clinical management of the cariostatic agents was not a major barrier to its wider use in Australia. Rather, attention should be directed toward the higher-level determinants as previously discussed.

### Strengths and Limitations

This article provides dental stakeholders’ perceptions of the barriers and facilitators to SDF implementation into Australia, which is an unheard perspective across the literature. One limitation of the study is the small sample size, which was influenced by challenges in recruitment engagement. While recruitment successfully included participants from more than 50% of Australian states and diverse clinical and professional roles, engagement through recruitment channels declined over time, despite multiple follow-up attempts. The ability to secure further participation beyond the initial sample proved difficult due to factors such as time constraints, availability of potential participants, and willingness to engage in the study. Further, as data collection progressed, limited new insights emerged from interviews, indicating thematic saturation may have been reached. A decision was made to conclude recruitment given these recruitment challenges and the diminishing returns from additional interviews. However, a larger sample size with stakeholders from all states, as well as perspectives from a policy level, could have further strengthened our understanding of the current state of SDF in Australia’s dental health system. Recruitment relied on professional networks, which may have limited the inclusion of diverse perspectives and experiences.

## Conclusion

There are barriers and facilitators to SDF implementation into Australia that exist across themes of policy, teaching and exposure, manufacturer, and clinical settings. The lack of teaching and exposure to SDF in the undergraduate dental setting and the lack of detailed or standardized Australian guidelines on use have prevented clinician awareness and confidence to use SDF. Without sufficient knowledge, there is hesitancy for its use with potential perpetuation of the stigma of SDF, limiting its use as a tertiary prevention strategy for managing dental caries. There should be prioritization of gaining TGA approval for caries use to address legal concerns; this would facilitate the development of educational training for clinicians and public programs for patients. The manufacturer plays a key role in pushing for TGA approval for caries and the marketing and design of the product available in Australia. For successful implementation of SDF into Australia, there must be collaboration and planning surrounding its policies, the manufacturer, education, and consideration of the clinical setting.
